# IL-1β and IL-17 in cutaneous lupus erythematous skin biopsies: could immunohistochemicals indicate a tendency towards systemic involvement?^☆^

**DOI:** 10.1016/j.abd.2023.02.007

**Published:** 2023-09-30

**Authors:** Barbara Hartung Lovato, Leticia Fogagnolo, Elemir Macedo de Souza, Larissa Juliana Batista da Silva, Paulo Eduardo Neves Ferreira Velho, Maria Leticia Cintra, Fernanda Teixeira

**Affiliations:** a Department of Pathology, Faculdade de Ciências Médicas, Universidade Estadual de Campinas, Campinas, SP, Brazil; b Department of Dermatology, Faculdade de Ciências Médicas, Universidade Estadual de Campinas, Campinas, SP, Brazil

**Keywords:** Cutaneous lupus erythematosus, Cytokines, IL-17, Immunohistochemistry, Interleukin-1beta, Systemic lupus erythematosus

## Abstract

**Background:**

Only a fraction of patients with cutaneous lupus erythematosus (CLE) will eventually progress toward systemic disease (SLE).

**Objective:**

To find inflammatory biomarkers which could predict the progression of cutaneous lupus erythematosus (CLE) into systemic lupus erythematosus (SLE) using immunohistochemical (IHC) assays.

**Methods:**

Immunohistochemical markers for cytotoxic, inflammatory, and anti-inflammatory responses and morphometric methods were applied to routine paraffin sections of skin biopsies, taken from lesions of 59 patients with discoid lupus, subacute lupus, and lupus tumidus. For the diagnosis of SLE, patients were classified by both the American College of Rheumatology (ACR-82) and the Systemic Lupus International Collaborating Clinics (SLICC-12) systems.

**Results:**

Skin samples from CLE/SLE + patients presented higher expression of IL-1β (ARC-82: p = 0.024; SLICC-12: p = 0.0143) and a significantly higher number of cells marked with granzyme B and perforin (ARC: p = 0.0097; SLICC-12: p = 0.0148). Biopsies from CLE/SLE- individuals had higher expression of IL-17 (ARC-82: p = 0.0003; SLICC-12: p = 0.0351) and presented a positive correlation between the density of granzyme A + and FoxP3+ cells (ARC-82: p = 0.0257; SLICC-12: p = 0.0285) and CD8+ cells (ARC-82: p = 0.0075; SLICC-12: p = 0.0102), as well as between granulysin-positive and CD8+ cells (ARC-82: p = 0.0024; SLICC-12: p = 0.0116).

**Study limitations:**

Patients were evaluated at a specific point in their evolution and according to the presence or not of systemic disease. The authors cannot predict how many more, from each group, would have evolved towards SLE in the following years.

**Conclusions:**

In this cohort, immunohistochemical findings suggested that patients with a tendency to systemic disease will show strong reactivity for IL-1β, while those with purely cutaneous involvement will tend to express IL-17 more intensely.

## Introduction

Among the criteria listed by both the American College of Rheumatology (ACR-82) and the Systemic Lupus International Collaborating Clinics (SLICC-12) for the diagnosis of Systemic lupus erythematosus (SLE), four are related to changes in the skin and mucous membranes.^1^, ^2^ Cutaneous lupus erythematosus (CLE) is one of the most conspicuous findings, but only a fraction of patients with CLE will eventually develop SLE: among patients with discoid lupus erythematosus (DLE), the most common subtype of CLE, the frequency of progression to SLE ranges from 0% to 28%.^3^ Characteristics that lead the physician to suspect that this may happen include the number and severity of skin lesions, the titer of antinuclear antibodies, and the presence of systemic symptoms at baseline.^4^ The possibility of progression to SLE causes anxiety in patients with cutaneous lupus. It is important, then, to try to determine which patients are at higher risk of progression to systemic disease.

Previous research has shown the importance of pro-inflammatory and cytotoxic pathways in lupus erythematosus genesis, usually comparing CLE subtypes. Nevertheless, few studies have focused on differences between CLE/SLE + e and CLE/SLE- patients. Our objective was to find inflammatory biomarkers which could predict the CLE progression into SLE. For this purpose, the authors used Immunohistochemical (IHC) assays in CLE skin biopsies (Supplementary Table 1).

**Table 1:** Demographic and clinical data of patients, according to CLE subtypes and the presence of SLE.

CLE subtype	ARC-82		SLICC-12	
	SLE+	SLE-	SLE+	SLE-
	n (%)	n (%)	n (%)	n (%)
DLE (n = 21)	**4 (19)**	**17 (80.9)**	**4 (19)**	**17 (80.9)**
Mean age, years	31	45	31	45
Sex, Female (%)	100	82.3	100	82.3
Positive ANA (%)	25	0	25	0
Anti-DNA (%)	75	0	75	0
Positive anti-SM (%)	25	0	25	0
SAL (n = 17)	**12 (70.5)**	**5 (29.4)**	**8 (47.05)**	**9 (52.9)**
Mean age, years	40	40	39	41
Sex, Female (%)	75	100	75	89
Positive ANA (%)	100	60	100	78
Positive anti-DNA (%)	42	0	51	0
Positive anti-SM (%)	17	0	25	0
LT (n = 21)	**4 (19.04)**	**17 (80.9)**	**4 (19.04)**	**17 (80.9)**
Mean age, years	40	35	39	35
Sex, Female (%)	100	94	100	94
Positive ANA (%)	100	30	100	30
Positive anti-DNA (%)	25	0	25	0
Positive anti-SM (%)	0	0	0	0

CLE, Cutaneous Lupus Erythematosus; DLE, Discoid CLE; SAL, Subacute CLE; LT, CLE Tumidus; ARC, American Rheumatology College; SLICC, Systemic Lupus International Collaborating Clinics; SLE, Systemic Lupus Erythematosus.

## Methods

Skin biopsies from 59 patients with CLE seen at the Dermatology Outpatient Center between 1995 and 2012 were included if: 1) Complete medical records were available, 2) The diagnosis was confirmed by clinical and histological examination, including direct immunofluorescence, when indicated, and 3) Enough remaining paraffin-embedded tissue was found for new sections. These were treated by conventional immunohistochemistry methods, using Polymers (Kit Novolink, Novocastra Laboratories, Newcastle-upon-Tyne, United Kingdom) or the streptavidin-biotin-horseradish peroxidase method (LSAB-HRP, code 0609, Dako Cytomation, Glostrup, Denmark). The lymph node, appendix and tonsil sections were used as controls. Primary antibodies, recovery method and detection system employed are detailed in Supplementary Table 2.

**Table 2:** Immunohistochemical differences between CLE/SLE + and CLE/SLE- patients, according to ARC-82 and SCLICC classifications.

ARC-82			
	CLE/SLE+ (n = 20)	CLE/SLE- (n = 39)	p-value
IL-1β (epidermal cells), n (%)			0.024
1 (mild)	2 (10)	5 (12.8)	
2 (moderate)	2 (10)	16 (41)	
3 (intense)	16 (80)	18 (46.2)	
IL-1β (interstitial)			<0.05
0	0	3	
1 (mild)	3	10	
2 (moderate)	16	23	
3 (intense)	1	3	
IL-17 (epidermal cells)			<0.05
1 (mild)	0	5	
2 (moderate)	5	8	
3 (intense)	15	26	
IL-17 (interstitial)			0.0003
1 (mild)	17 (85)	14 (35.9)	
2 (moderate to intense)	3 (15)	25 (64.1)	
% of CD56 immunostained cells in 3 fields (mean ± SD)		0.013	
	7.2 ± 3.1	5.6 ± 3.9	
% of ICAM immunostained cells in 3 fields (mean ± SD)		0.0095	
	47.5 ± 6.7	41.8 ± 7.7	

SCLICC			
	CLE/SLE+ (n = 16)	CLE/SLE- (n = 43)	p-value
Il-1β (epidermal cells)			0.0143
1 (mild)	0 (0)	7 (16.3)	
2 (moderate)	2 (12.5)	16 (37.2)	
3 (intense)	14 (87.5)	20 (46.5)	
IL-1β (interstitial)			<0.05
1 (mild)	2 (12.5)	14 (32.6)	
2 (moderate)	13 (81.3)	26 (60.5)	
3 (intense)	1 (6.3)	3 (7)	
IL-17 (epidermal cells)			<0.05
1 (mild)	0	0	
2 (moderate)	14 (87.5)	33 (76.7)	
3 (intense)	2 (12.5)	10 (23.3)	
IL-17 (interstitial)			0.0351
1 (mild)	12 (75)	19 (44.2)	
2 (moderate to intense)	4 (25)	24 (55.8)	

CLE, Cutaneous Lupus Erythematosus; DLE, Discoid CLE; SAL, Subacute CLE; LT, CLE tumidus; ARC, American Rheumatology College; SLICC, Systemic Lupus International Collaborating Clinics; SLE, Systemic Lupus Erythematosus.

Biomarkers were used to assess cytotoxic, inflammatory and anti-inflammatory responses. For the analysis of ICAM-1, CD4, CD8, CD25, CD56, Granzyme A, Granzyme B, Granulysin, Perforin and FOXP3, three (×400) fields with the highest density of inflammatory cells and no artifacts were chosen. Their digital images were acquired and analyzed with the aid of the Image J software (Java-based image processing program, Wayne Rasband, NIH, Bethesda, USA, available at http://rsb.info.nih.gov/ij). Both the immunostained cells and the others, present in the same field, were counted. The expression of each marker was then defined as the ratio between the number of immunostained cells and the total number of cells counted. For the IL-6, IL-1β, IL-17, IL-18 and IL-10 markers, the authors used the analysis method described by Popovic et al.^5^

The median age of the 59 patients was 39 years; 55 were female (93.2%); 21 had discoid lupus (DLE), 21 had lupus tumidus (LT) and 17 had subacute lupus (SAL). Nineteen percent of patients with DLE and LT had clinical features of systemic involvement, as classified by both ARC-82 and SLICC-12 criteria. Regarding patients with SAL, 47% were labeled as systemic according to the SLICC-12 classification; in contrast, 70.5% of the same patients were classified as having systemic involvement by the ARC-82 guidelines (Table 1). This difference occurred because SLICC-12 does not include photosensitivity as a criterion, while ARC-82 does not consider synovitis and positive Coombs test criteria.

For statistical analysis, the authors used SAS 9.0® software. Exploratory data analysis was performed by summary measures (mean, standard deviation, minimum, median, maximum, frequency, and percentage). The groups (SLE + and SLE- patients) were compared by Fisher's exact test, Chi-Square or Mann-Whitney. The correlation between numerical variables was assessed using Spearman's coefficient. The adopted level of significance was 5%.

The study was approved by the institution's ethics committee (# 1214/2010), in which all the Declaration of Helsinki principles were observed.

## Results

In all samples, the cells were immunostained by all antibodies (Supplementary Table 1), but for some markers, there were statistically significant differences between CLE/SLE + and CLE/SLE- groups, as detailed in Table 2.

**Figure 1:**
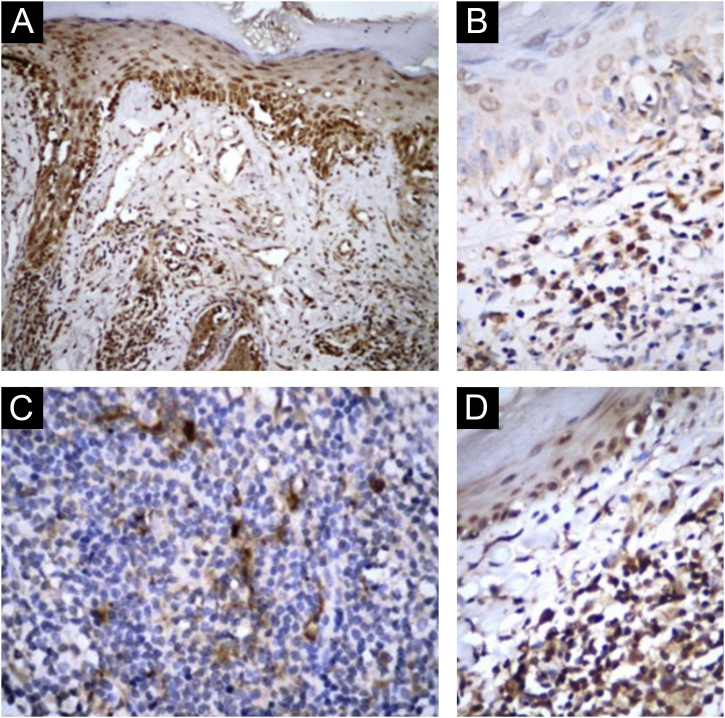
Immunohistochemical findings: (A‒B) IL-1β. (C‒D) IL-17. A‒C, SLE patients; B‒D, patients with purely cutaneous lupus erythematosus lesions (Original magnification: A = ×100; B‒D = ×400).

In CLE/SLE + patients, as classified by both systems, there was greater expression of IL-1β (Fig. 1 A and B; ARC-82: p = 0.024; SLICC-12: p = 0.0143), compared to CLE/SLE- patients. Regarding cytotoxic T response markers, CLE/SLE + patients had a significantly higher number of cells marked with Granzyme B and Perforin (Table 3) than CLE/SLE- patients (ARC: p = 0.0097; SLICC-12: p = 0.0148).

**Table 3:** Immunohistochemical differences in Spearman correlation between CLE/SLE + and CLE/SLE- patients, according to ARC-82 and SCLICC classifications.

	CLE/SLE+	
Variable	P (ARC-82), n = 20	P (SLICC-12), n = 16
*Spearman correlation*		
Perforin and granzyme B correlation	0.0097	0.0148

	CLE/SLE-	
Variable	P (ARC-82), n = 39	P (SLICC-12), n = 43
*Spearman correlation*		
FoxP3 and granzyme A correlation	0.0257	0.0285
CD8 and granzyme A correlation	0.0075	0.0102
CD8 and granulysin correlation	0.0024	0.0116

Patients classified by both systems as CLE/SLE- had higher expression of IL-17 (Fig. 1 C and D; ARC-82: p = 0.0003; SLICC-12: p = 0.0351). In addition, there was a positive correlation between the density of Granzyme A + cells, both with that of FoxP3+ (ARC-82: p = 0.0257; SLICC-12: p = 0.0285) and CD8+ cells (ARC-82: p = 0.0075; SLICC-12: p = 0.0102), as well as between Granulysin-positive and CD8+ cells (ARC-82: p = 0.0024; SLICC-12: p = 0.0116). There was also a negative correlation between the density of Granulysin-positive cells and the following SLE criteria (SLICC-12): synovitis (p = 0.0466), hypocomplementemia (p = 0.0408) and anti-SM antibody (p = 0.0252).

## Discussion

Macrophages, among other cells, secrete IL-1β through different stimuli, especially IFN-α. The importance of a pro-inflammatory response stimulated by IFN type I, particularly IFN-α, in SLE + patients, is supported by clinical parameters, such as systemic symptoms (such as fever and fatigue), skin biopsies and autopsy findings.^6^, ^7^, ^8^, ^9^ Wenzel et al. showed that patients with disseminated discoid lesions or SAL had increased serum levels of IFN type I, which may indicate an increased risk of developing systemic disease.^10^ In the skin, IFN can stimulate the synthesis and overexpression of cytokine receptors that promote the inflow of immune cells and the development of lesions.^11^ In our sample, this pro-inflammatory response in CLE/SLE + individuals was identified by the expression of IL-1β in skin biopsies. Zhou et al. found increased serum levels of IL-1β in patients with SLE evaluated by cytometric bead array,^12^ showing dysregulated T-cell activity.

In contrast, in CLE/SLE- patients, the inflammatory response marker was IL-17. Studies examining the role of IL-17 in lupus erythematosus, to investigate the efficacy of anti-IL-17 immunobiological medications, reported an increase in IL-17 expression in SAL and DLE lesions and in unaffected skin of SLE + patients, compared to healthy controls.^8^, ^13^ In our sample, IL-17 expression was significantly higher in CLE/SLE- patients, suggesting that anti-IL-17 drugs could be effective for CLE lesions.

In CLE/SLE + patients with any CLE subtype, the authors identified a cytotoxic T response by increased expression of Granzyme B and Perforin granules, necessary for penetration into target cells. Granzyme B is strongly expressed in healing CLE lesions and significantly increased when compared to SAL and healthy controls.^14^ Grassi et al. demonstrated that the expression of Granzyme B + cells was more frequent in skin biopsies from SLE + patients, compared to patients with purely CLE, suggesting that Granzyme B is a key cytotoxicity marker in SLE + individuals.^15^ Its role in the genesis of multiple SLE manifestations is also supported by the higher levels of soluble serum Granzyme B in SLE + patients.^16^

In CLE/SLE- patients, the positive correlation between CD8+, Granzyme A+, and Granulysin + cell density highlights the importance of cytotoxicity in the pathogenesis of skin lesions. Previous studies have shown a predominance of CD8+ cells in the lesions of patients with DLE, while in other skin subtypes more frequently associated with systemic disease, such as SAL, there is a predominance of CD4+ cells.^17^, ^18^ Other studies emphasized only the role of Granzyme B in CLE lesions, without considering the possible association with systemic disease.^14^, ^18^ Granzyme A associated with IL-17 expression was previously described in psoriatic lesions,^19^ which may indicate, in a psoriatic environment, a predominant role in the maintenance of inflammation, rather than cytotoxicity.

The patients in our sample were evaluated at a specific point in their evolution when their symptoms, signs and laboratory results could classify them as CLE/LES- or CLE/LES + patients. The authors cannot predict how many more, from each group, would have evolved toward systemic disease in the next five, or ten years. The authors believe that the probability of developing systemic lupus is greater in the SAL group, and the authors cannot safely exclude the possibility that the differences found may only reflect those between DLE/LT and SAL. It is important to highlight that the authors did not perform comparative analysis between the subgroups of CLE, since our main objective was to evaluate differences between CLE/SLE + and CLE/SLE- patients, regardless of the subtype of CLE.

## Conclusion

In this sample, immunohistochemical findings suggest that patients with a tendency to systemic disease will show strong reactivity to IL-1β, while those with purely cutaneous involvement will tend to express IL-17 more intensely. The findings can help, together with clinical data, to identify CLE patients more likely to progress to systemic disease.

## Financial support

This work was supported by the São Paulo State Research Support Foundation (FAPESP) – [Process # 11/50037-2 and # 2016/153362].

## Authors' contributions

Bárbara Hartung Lovato: Conception and planning of the study; critical review of the literature; obtaining, analysis, and interpretation of the data; elaboration and writing of the manuscript; critical review of the manuscript; approval of the final version of the manuscript.

Letícia Fogagnolo: Conception and planning of the study; critical review of the literature; obtaining, analysis, and interpretation of the data; critical review of the manuscript; approval of the final version of the manuscript.

Elemir Macedo de Souza: Conception and planning of the study; critical review of the manuscript.

Larissa Juliana Batista da Silva: Critical review of the literature; obtaining, analysis, and interpretation of the data; elaboration and writing of the manuscript.

Paulo Eduardo Neves Ferreira Velho: Critical review of the literature; obtaining, analysis, and interpretation of the data; critical review of the manuscript.

Maria Leticia Cintra: Conception and planning of the study; critical review of the literature; obtaining, analysis, and interpretation of the data; elaboration and writing of the manuscript; critical review of the manuscript; approval of the final version of the manuscript.

Fernanda Teixeira: Critical review of the literature; critical review of the manuscript; approval of the final version of the manuscript.

## Conflicts of interest

None declared.
