# Supplementary material for: IL-1β and IL-17 in cutaneous lupus erythematous skin biopsies: could immunohistochemicals indicate a tendency towards systemic involvement?
Source: An Bras Dermatol. 2023 Sep 30;99(1):66–71. doi: 10.1016/j.abd.2023.02.007 (PMC10964360; doi:10.1016/j.abd.2023.02.007)
Supplement: Supplementary file 1 [file mmc1.docx]

**ABD-D-22-00537_ Supplementary Material**

**Supplementary Table 1** Antibodies, target, staining pattern and site.

| **Antibody** | **Target** | **Staining pattern** | **Site** |
| --- | --- | --- | --- |
| CD4 | T-helper cells, but occasionally also macrophages and dendritic cells | Membranous and cytoplasmic | Υ |
| FoxP3 | CD4+CD25+ regulatory T-cells | Nuclear |  |
| CD25 | CD4+ regulatory T-cells but occasionally also mature Natural Killer (NK) cells, recent thymic emigrants, eosinophils, and basophils | Membranous and cytoplasmic |  |
| CD8 | Suppressor/cytotoxic T-cells |  |  |
| CD56 | NK cells, but occasionally also alpha beta T-cells, gamma delta T-cells, and dendritic cells |  |  |
| Cytotoxic cells^a^ | Cytotoxic T and NK cells, but occasionally also granulocytes | Cytoplasmic |  |
| ICAM-1 | Endothelial, epithelial, and immune cells | Membranous | ∞ |
| IL-1β | Epithelial and mesenchymal cells | Cytoplasmic and interstitial | µ |
| IL-6 |  |  | € |
| IL-10 |  |  | £ |
| IL-17 |  |  | Ω |
| IL-18 |  |  | ¥ |
| TNF-α |  |  | π |

^a^ Ganzymes, granulysin, and perforin.

Υ, Inflammatory cells in the dermis, epidermis, and appendages; ∞, Υ and endothelium; µ, Keratinocytes, sweat epithelium, inflammatory, endotelial cells, dermal interstitium; €, Inflammatory and dendritic cells, fibrous sheath of hair follicle and papilla cells of follicular bulb; dermal interstitium; £, Keratinocytes, sweat epithelium, inflammatory, dendritic and endotelial cells; Ω, Keratinocytes, inflammatory cells, and dermal interstitium; ¥, Follicular inner sheath, sebocytes, perifollicular fibrous sheath dendritic cells, and inflammatory cells; π, epidermis, macrophages, dendritic cells.

**Supplementary Table 2** Antibodies, clones, dilution, recovery method and detection system.

| **Primary antibody** | **Clone** | **Dilution** | **Antigen recovery (steam – pressure cooker)** | **Detection System** |
| --- | --- | --- | --- | --- |
| CD4 | SP35; Spring Bioscience, Pleasanton, California, EUA | 1:100 | Citrate, pH 6.0 | Novolink. |
| CD8 | M7103, Dako, Glostrup, Dinamarca | 1:100 | Citrate, pH 9.0 | Novolink. |
| CD25 | 4C9; Cell Marque, Rocklin, California, EUA | 1:100 | Tris-EDTA, pH 9.0 | Novolink |
| CD56 | M7304, Dako, Glostrup, Dinamarca | 1:150 | Citrate, pH 6.0 | Novolink. |
| FOXP3 | PCH 101, eBioscience, San Diego, California, EUA | 1:200 | Tris- EDTA, pH 9.0 | Novolink. |
| Granzyme A | GA6; Santa Cruz Biotechnology, Dallas, Texas, EUA | 1:60 | Citrate, pH 6.0 | Novolink |
| Granzyme B | 11F1; Novocastra, Newcastle-upon-Tyne, Reino Unido | 1:50 | Tris-EDTA, pH 9.0 | Novolink |
| Granulysine | RJT48; Novocastra, Newcastle-upon-Tyne, Reino Unido | 1:50 | Tris-EDTA, pH 9.0 | Novolink |
| ICAM-1 | G5, Santa Cruz Biotechnology, Dallas, Texas, EUA | 1:8000 | Citrate, pH 6.0 | Novolink |
| IL-1β | H-153; Santa Cruz Biotechnology, Dallas, Texas, EUA | 1:40 | Tris-EDTA, pH 9.0 | LSAB-HRP |
| IL-6 | MQ2-13A5; Biolegend, San Diego, California, EUA | 1:500 | Citrate, pH 6.0 | Novolink |
| IL-10 | AF-217-NA; R&D Systems, Minneapolis, Minnesota, EUA | 1:50 | Tris-EDTA, pH 9.0 | LSAB-HRP |
| IL-17 | H-132; Santa Cruz Biotechnology, Dallas, Texas, EUA | 1:50 | Tris-EDTA, pH 9.0 | Novolink |
| IL-18 | N-19; Santa Cruz Biotechnology, Inc, Dallas, Texas, EUA | 1:40 | Tris-EDTA, pH 9.0 | LSAB-HRP |
| Perforine | 5B10; Novocastra, Newcastle-upon-Tyne, Reino Unido | 1:30 | Tris-EDTA, pH 9.0 | Novolink |
| TNF-α | AF-210-NA; R&D Systems, Minneapolis, Minnesota, EUA | 1:50 | Citrate, pH 6.0 | LSAB-HRP. |
